# An Automatic Image Processing Method Based on Artificial Intelligence for Locating the Key Boundary Points in the Central Serous Chorioretinopathy Lesion Area

**DOI:** 10.1155/2023/1839387

**Published:** 2023-02-10

**Authors:** Jianguo Xu, Jianxin Shen, Cheng Wan, Zhipeng Yan, Fen Zhou, Shaochong Zhang, Weihua Yang

**Affiliations:** ^1^College of Mechanical and Electrical Engineering, Nanjing University of Aeronautics and Astronautics, Nanjing 210016, China; ^2^College of Electronic and Information Engineering, Nanjing University of Aeronautics and Astronautics, Nanjing 210016, China; ^3^The Affiliated Eye Hospital of Nanjing Medical University, Nanjing 210029, China; ^4^Shenzhen Eye Hospital, Jinan University, Shenzhen 518040, China

## Abstract

Accurately and rapidly measuring the diameter of central serous chorioretinopathy (CSCR) lesion area is the key to judge the severity of CSCR and evaluate the efficacy of the corresponding treatments. Currently, the manual measurement scheme based on a single or a small number of optical coherence tomography (OCT) B-scan images encounters the dilemma of incredibility. Although manually measuring the diameters of all OCT B-scan images of a single patient can alleviate the previous issue, the situation of inefficiency will thus arise. Additionally, manual operation is subject to subjective factors of ophthalmologists, resulting in unrepeatable measurement results. Therefore, an automatic image processing method (i.e., a joint framework) based on artificial intelligence (AI) is innovatively proposed for locating the key boundary points of CSCR lesion area to assist the diameter measurement. Firstly, the initial location module (ILM) benefiting from multitask learning is properly adjusted and tentatively achieves the preliminary location of key boundary points. Secondly, the location task is formulated as a Markov decision process, aiming at further improving the location accuracy by utilizing the single agent reinforcement learning module (SARLM). Finally, the joint framework based on the ILM and SARLM is skillfully established, in which ILM provides an initial starting point for SARLM to narrow the active region of agent, and SARLM makes up for the defect of low generalization of ILM by virtue of the independent exploration ability of agent. Experiments reveal the AI-based method which joins the multitask learning, and single agent reinforcement learning paradigms enable agents to work in local region, alleviating the time-consuming problem of SARLM, performing location task in a global scope, and improving the location accuracy of ILM, thus reflecting its effectiveness and clinical application value in the task of rapidly and accurately measuring the diameter of CSCR lesions.

## 1. Introduction

**Figure 1:**
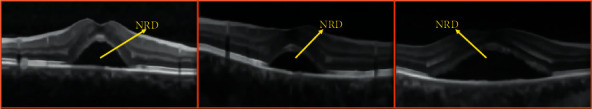
The typical lesion characteristic of CSCR: NRD.

CSCR is a common fundus macular disease, which causes the visual object to be deformed, darkened, or become smaller and is one of the factors affecting human visual health. But its pathogenesis is still unknown in ophthalmology. In recent years, some scholars have put forward new theories on the pathogenesis of CSCR, such as the theory of choroidal dysfunction and the theory of retinal pigment epithelium dysfunction, which have explained the pathogenesis of CSCR to a certain extent and appropriately promoted human cognition of the fundus disease. This macular disease is mostly seen in young men aged 30 to 50 and is typically characterized by neurosensory retinal detachment (NRD, as shown in Figure 1) with or without pigment epithelium detachment (PED) [1, 2]. Although the vision of some patients may recover spontaneously within a few months without any intervention, it is still difficult for some patients to recover to normal vision without surgery or drugs in a short time. In general, the main active interventions for the treatment of CSCR are laser surgery and drugs. No matter which method is adopted, it is essential and critical to carry out effective quantitative monitoring of the CSCR lesion area, which lays a foundation for timely obtaining the disease information and then assisting ophthalmologists to more objectively evaluate the severity of this disease and the efficacy of the corresponding treatment plan and also provides a basis for better formulating the follow-up treatment scheme.

At the moment, the monitoring parameters of CSCR mainly include the central macular thickness (CMT), best corrected visual acuity (BCVA), maximum height, and diameter of CSCR lesion area. In addition, the CSCR lesion area is also an important parameter, and its direct segmentation and indirect detection methods have been carried out by many researchers [3–9]. A fully convolutional neural network was built for the automatic segmentation of subretinal fluid, and with the help of shrinking and expanding network structure, an average dice rate of 0.91 was obtained [3]. To deal with the large variations of the locations and shapes of CSCR lesion and the low contrast of Bruch membrane areas, Xue et al. [4] proposed a deep ensemble neural-like P system that integrated the strengths of deep convolutional neural networks and the spiking neural P system and achieved a maximum average dice rate of 0.97, which showed great potential in actual application. Wu et al. [5] presented a two-stage scheme consisting of detecting fluid-associated abnormalities by using thickness map prior and segmenting the subretinal fluid by using the fuzzy level set with a spatial smoothness and was beneficial for the automatic quantification of lesion area. Similar to [3], an end-to-end pipeline [6] inspired by the SegNet neural network was adopted for the identification and segmentation of CSCR fluid regions, which facilitated a more complete analysis of CSCR. Based on loosely coupled level sets, Novosel et al. [7] raised a locally-adaptive approach for the segmentation of the fluid and the interfaces between retinal layers, and a dice coefficient for fluid segmentation of 0.96 was acquired, which revealed a great potential in quantifying the CSCR lesion area. Moreover, Zhen et al. [8] tried to detect CSCR based on the deep learning architecture and color fundus images. However, this method cannot describe CSCR lesions in detail, so it is not conducive to the monitoring of the disease. A commendable segmentation model combining the U-Net and generative adversarial network was ingeniously constructed by Yoo et al. [9]. To the best of our knowledge, this framework was the first time to achieve the segmentation of CSCR lesions in the color fundus images by developing a cascaded network, which is of great significance for quantitative monitoring of CSCR by virtue of conventional fundus image examination. In addition to the previous direct segmentation schemes, there are also some indirect detection methods [10, 11]. Syed et al. [10] constructed a support vector machine (SVM) classifier-based model for the automated diagnosis of CSCR. Specifically, they established a feature vector with a length of 8 based on retinal thickness and cyst space cavity to guide the classifier to learn proper weights for judging the disease category. A similar idea was also designed by Khalid et al. [11], where the difference was that 9 extracted features and more testing samples were adopted to train the classifier for making more accurate judgments on the type of retinal diseases. In these schemes, the feature descriptors of the CSCR lesion area are firstly established by applying the feature engineering technique, and then, the classifier is trained by using the feature vectors to construct the lesion detection model. Since such schemes require detailed digital description of the lesions, professional cognition of the characteristics of the lesions is crucial.

Besides, the fluid segmentation of other fundus diseases also provides a reference for the area quantification method of CSCR lesion [12–17]. To detect three-dimensional retinal fluid (i.e., symptomatic exudate-associated derangements), Xu et al. [12] developed a novel voxel classification-based approach using a layer-dependent stratified sampling strategy, and this approach performed well in dealing with the class imbalance issue. By combining the squeeze-and-excitation blocks and the U-shape network, Chen et al. [13] put forward a structure called SEUNet to segment fluid regions in the age-related macular degeneration (AMD) and supplied an effective for fluid segmentation. Based on graph shortest paths and neutrosophic transformation, a fully-automated segmentation method was designed for the accurate segmenting of diabetic macular edema (DME) biomarkers so as to provide a quantitative measure for DME diagnosis [14]. Alsaih et al. [15] employed four wide-spread deep learning models for the segmentation of three retinal fluids in AMD and explored how the patch-based technique pushes the performance of deep learning-based models, which was conducive to the improvement of such scheme. Lu et al. [16] presented a deep learning-based method for segmenting multiclass retinal fluids. Different from the common deep learning schemes, this method introduced the random forest classifier in postprocessing to reduce the over segmentation problem in the independent network model. Hassan et al. [17] also constructed a deep learning-based segmentation network integrating the atrous spatial pyramid pooling module, residual module, and inception module to segment multiclass retinal fluids and brought a considerable gain in efficiency.

The previous direct segmentation pipelines or indirect detection methods undoubtedly enrich the research ideas of the automatic quantification scheme of the CSCR lesion area, which is of great significance for the precise treatment of this eye disease. Nevertheless, the tediousness of the pixel-level annotation task in deep learning-based segmentation method and its potential defects of insufficient generalization ability, the strong dependence of feature engineering on professional experience in classical machine learning-based detection way, and the low accuracy and weak adaptability in traditional image processing-based segmentation plan may restrict the wide application of the previous methods in the quantification task of CSCR lesion area to a certain extent. It has to be said that the lesion diameter measurement scheme [18] based on locating key boundary points does appropriately alleviate the previous situations, but the gradient-based correction module (GBCM) in this scheme relies on setting appropriate threshold parameters and is sensitive to the position of the starting point provided by ILM.

**Figure 2:**
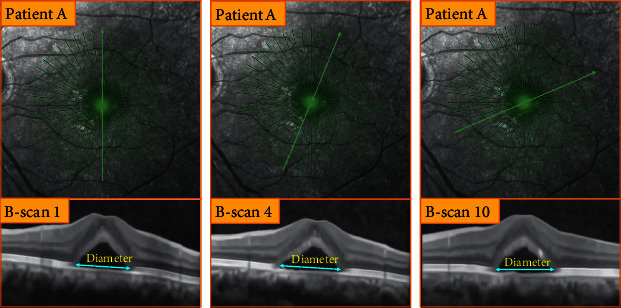
The diameter difference of CSCR lesion area in B-scan images under different frames.

Considering the limitations and advantages of the previous methods, as well as the challenges of diameter measurement caused by the diameter differences of CSCR lesion areas in different frames (as shown in Figure 2), this paper constructs an automatic image processing method (i.e., a joint framework) based on artificial intelligence for rapidly and accurately measuring diameter of CSCR lesion area from the perspective of locating key boundary points in the CSCR lesion area. The details are as follows: (1) In the first step, the multitask learning-based ILM is appropriately adjusted and used for rapid location of key boundary points in the CSCR lesion area, laying the foundation for subsequent accuracy improvement. (2) In the second step, the location task is described as MDP, in which the single agent aims to explore and lock the key boundary points in the CSCR lesion area through continuous interaction with the image environment. (3) The joint framework based on ILM and SARLM is skillfully designed to make up for the defect of low generalization of ILM by employing the unique exploration ability of agent in SARLM and to narrow the active region of single agent by providing the initial starting point for SARLM through the ILM. (4) In the fourth step, extensive and in-depth experiments are carefully carried out to prove and analyze the effectiveness and feasibility of the joint framework in the key boundary point location task of CSCR lesion area and its application effect in the lesion diameter measurement.

The structure of the remaining part is as follows: Section 2 describes the related works of multitask learning and single agent reinforcement learning.  Section 3 explains the implementation details of our proposed method.  Section 4 shows the results and discussions.  Section 5 concludes the research work.

## 2. Materials and Related Methods

### 2.1. Materials

The CSCR source images used in the experiments are provided by the cooperative eye hospitals, and the patients' privacy information has been desensitized carefully. The annotation task of all the CSCR B-scan images is jointly completed and reviewed by professional ophthalmologists and relevant academic personnel. After the conventional data augmentation operations, the number of image and annotation pairs in the dataset used for training reaches 3240. Additionally, to evaluate the effect of the joint framework in the testing dataset, a total of 25 patient-level data, including 912 OCT B-scan images, are introduced into this process.

### 2.2. Related Methods

#### 2.2.1. Multi-Task Learning

As one of the artificial intelligence technologies, multitask learning [19] is a learning paradigm that improves generalization ability of the convolutional neural network model by using the domain information contained in the training signals of related tasks as an inductive bias, which has been extensively applied in downstream tasks such as object detection, target classification, and semantic segmentation [20–22]. Meanwhile, this paradigm also shines brightly in various medical image processing tasks [18, 23–25]. For assisting the diameter measurement of the CSCR lesion area, the multitask learning paradigm was introduced into the key boundary point location task for the first time [18], enabling the rapid locking of the relevant coordinates. To obtain a robust retinal disease grading model, Ju et al. [23] extracted additional monitoring signals from various sources by using multitask learning and achieved a significant improvement. A new canonical correlation analysis model [24] combining the biologically meaningful structures with the multitask learning framework was designed to mine the shared representations in multimodal data, which experimentally demonstrated the potential of multitask learning. Additionally, this paradigm also performed well in improving the accuracy of glaucoma diagnosis [25]. By sharing most of the parameters of the segmentation layers and classification layers, the feature representation ability of the model for a given task is enhanced, and then a win-win situation is achieved.

#### 2.2.2. Reinforcement Learning

As a unique machine learning method to realize artificial intelligence, the reinforcement learning (RL) model has emerged in various scenes with its unique operating principle, in which the artificial agent obtains rewards and punishments through the continuous interaction with the environment [26] and then learns the optimal strategy for a given task. In particular, RL has shown satisfactory performance in various tasks in the field of medical image processing, such as registration, classification, and segmentation. In the registration task [27, 28], instead of directly optimizing an image matching metric, the goal of artificial agent was to find the best sequence of motion actions to achieve the best alignment between images. In the classification task [29], the agent cropped the appropriate patch on the original image through hard attention mechanism and updated the cropping strategy with the feedback of the classification network, so as to achieve better classification accuracy of breast cancer. In the segmentation task [30], the process of lymph node segmentation was completed by the interaction of two networks, where the decision network provided the target bounding box for the segmentation network, and the output of the segmentation network guided the policy network to make better strategies. Moreover, RL has also been applied and performed well in landmark detection [31–34]. Different from the traditional machine learning schemes, in this kind of application, the object appearance and parameter search strategy are unified into a framework, in which the behaviour strategy of agent and the effective object feature representation are jointly learned to better achieve the given task.

The previous research explored and confirmed the feasibility and effectiveness of the application of multitask learning and RL in the corresponding scenes and also promoted the inspiration of our research ideas in this paper. The specific details will be shown in the following sections.

## 3. The Proposed Method

**Figure 3:**
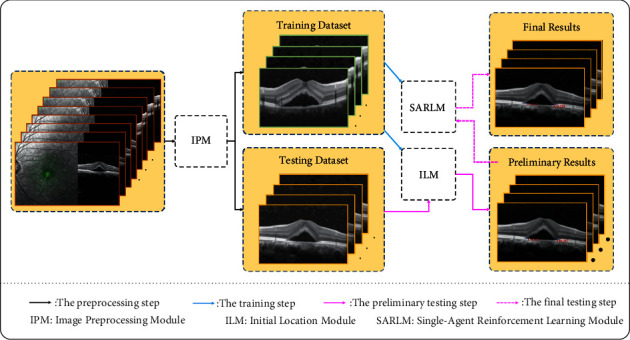
An overall flow chart.

As shown in Figure 3, it is the overall flow chart of the scheme proposed in this paper, including image preprocessing module (IPM), ILM, and SARLM. Firstly, IPM is employed to provide datasets for the subsequent training and testing steps of ILM and SARLM. Secondly, the operation of independently training ILM and SAILM based on the training dataset is carefully performed. Then, the testing images are input into the trained ILM model to obtain the preliminary results of key boundary points. Finally, the testing images and the corresponding location results are sent to SARLM to get the final results.

### 3.1. Motivation

Through the previous brief analysis, it can be clearly found that both the multitask learning and reinforcement learning have achieved a wide layout in various visual tasks and obtained gratifying results. In the previous applications, the multitask learning paradigm does improve the adaptability of the deep learning model to a certain extent. Nevertheless, the paradigm usually works independently in the downstream tasks, thus resulting in the generalization of the multitask model that is still affected by factors such as the volume of data and network structure. Although the RL model performs well in different scenarios, agents usually regard the global region of the input image as the interactive environment, which is bound to lead to a significant increase in the time cost and computing power required to complete the task. This cannot help but bring some thoughts to our research in this paper, that is, whether these two learning paradigms can be integrated to alleviate the above issues. On the one hand, ILM is used to realize the preliminary and rapid location of key boundary points to provide the initial starting point for the RL model, which in turn achieves the reduction of the active region of artificial agent. On the other hand, based on the unique exploration ability of agent in the RL model, the position of key boundary points is further adjusted on the basis of the initial location results of ILM in a local range. The specific implementation route and experimental results will be detailed in the following sections. It is the successful application and surprising achievements of these two learning paradigms in various visual tasks that encourage us to make further attempts in this key boundary point location task.

### 3.2. The Preprocessing Step

**Figure 4:**
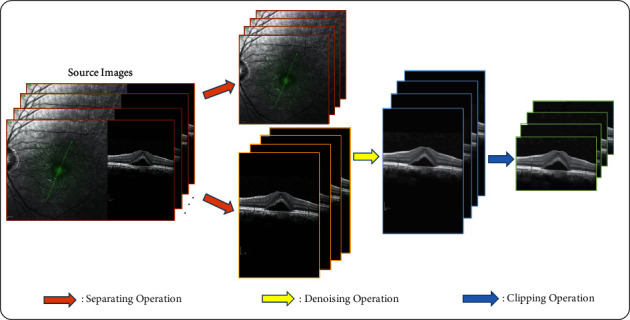
The image preprocessing process.

Due to the equipment and human factors, the quality and size of medical images initially obtained from the clinic are often unable to directly adapt to the downstream tasks, so the image preprocessing operation is particularly critical. In this paper, the source images acquired from the clinic are in a whole composed of the scanning laser ophthalmoscope (SLO) part and the OCT B-scan part, which cannot be directly applied to the key boundary points location tasks. In view of this, we use the separation operation designed in our previous work [18] to realize the separation of the previous parts. In addition, considering that the image size and speckle noise may interfere with the performance of both the ILM and SARLM, the clipping operation and the BM3D (Block-matching and 3D filtering [35])-based denoising operation are then applied to OCT B-scan images. The overall process of IPM is shown in Figure 4. After the image preprocessing, the size and quality of OCT B-scan images have been improved and then followed by image annotating step which is completed by professional ophthalmologists. Finally, the OCT B-scan image dataset used to locate the key boundary points in the CSCR lesion area is established, which paves the way for the follow-up work.

### 3.3. The Joint Framework

#### 3.3.1. ILM

**Figure 5:**
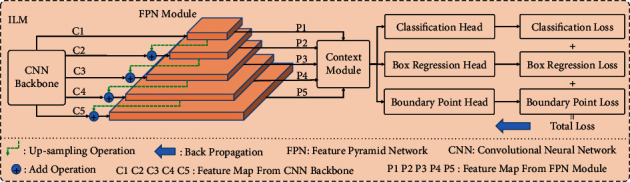
The overall framework of ILM.

Inspired by the excellent performance of the multitask model in face key point detection [36], we adjusted the architecture appropriately for the first time and introduced it into the key boundary point location scene in the CSCR lesion area [18], realizing another application test of this paradigm. In this paper, ILM continues to serve the task of initial location of key boundary points, and its specific composition is shown in Figure 5. The residual network [37] and MobileNet [38, 39] network are employed here as the CNN backbones to mine the background and nonbackground information contained in the OCT B-scan images, enabling the feature representation of CSCR lesion information. Specifically, we constructed the MobileNet with a width multiplier of 0.25 (i.e., MB1-0.25) [38] and its improved version (i.e., MB2-0.25) based on the inverted residual network [39], which are regarded as two kinds of backbone networks. Taking into account the network parameters and experimental conditions, resnet18 (i.e., R18), resnet34 (i.e., R34), and resnet50 (i.e., R50) are selected as another three kinds of backbone networks. The previous five backbone network structures are shown in Table 1. In addition, considering the capacity limitation in this paper, the FPN module [40], context module, and multitask loss module [41] will not be repeated further.

#### 3.3.2. SARLM

**Figure 6:**
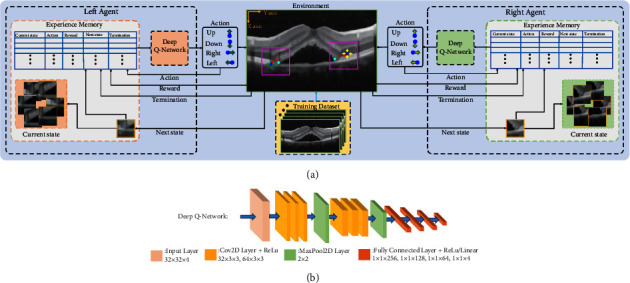
The overall framework of SARLM and deep Q-network: (a) SARLM; (b) deep Q-network.

As previously analyzed, RL has been popularized in various visual tasks; especially, its successful application in landmark detection promotes the proposal of our scheme. It should be noted that considering the distribution characteristics of key boundary points in this task and the time cost of agent interaction with the environment, this paper establishes SARLM to deal with the location task based on a single agent. The overall framework of SARLM is shown in Figure 6(a). Since the key boundary points are located on both sides of the CSCR lesion area, the SARLMs based on the left agent and the right agent are designed respectively. Although the structure of the two SARLMs is the same, the training process is carried out separately. Unlike the traditional machine learning scheme, the training samples required by SARLM are obtained through the continuous interaction between the agent and the environment, which are stored in the experience memory. The terms involved in the process are as follows:

(i) State: This term describes the surrounding environment including the location of the agent, which is mainly divided into the current state and the next state. In this task, in order to improve the operation efficiency of the agent, based on the initial location point provided by ILM, we first limit the active region of the agent to the purple square box (as shown in Figure 6(a)) with the size of 80. Then, with the location of the agent as the center, a square region with a size of 32 is cropped on the B-scan image as the state.
(ii) Action: This term refers to the moving direction of the agent in the environment, which is used to realize the interaction between the agent and the environment. In this paper, we set up four discrete actions, namely, up, down, left, and right, to control the agent to move in the corresponding direction with a step of one pixel, so as to achieve its exploration of the environment.
(iii) Reward: This term denotes the feedback of an agent after taking an action, aiming at evaluating whether the current action is conducive to the agent to achieve the given task. In the task of locating the key boundary points in the CSCR lesion area, the difference of the Euclidean distances between the agent and the target point before and after the action is regarded as reward. In addition, in order to avoid excessive *Q* value and obtain good conditional gradient, reward is clipped between −1 and 1 according to the common operation. The reward function is defined as follows:
  (1) $$\begin{array}{c} R=D\left(P_{i},P_{T}\right)-D\left(P_{i-1},P_{T}\right)\text{.} \end{array}$$
(iv) Policy: This term is a mechanism to determine the behavior of an agent. It is a mapping from the current state of the agent to the corresponding behavior taken by the agent. It defines various possible behaviors and corresponding probabilities of the agent in each state. In the key boundary point location task, the strategy is the behavior selection mechanism that enables the agent to reach the key boundary point of the CSCR lesion by a series of optimal actions. In the process of taking the optimal action, the agent can obtain the maximum cumulative reward.
(v) Termination: This term is used to define the stopping rules of agents in the training or testing stages, so as to prevent the agents from exploring and exploiting in the environment indefinitely. In this paper, in the training stage, we define the termination flag as true when the Euclidean distance between the agent and the target point is less than or equal to one pixel.
  (2) $$\begin{array}{c} T_{train}=\left\{\begin{array}{cc} \left\{\begin{array}{c} True,ifD\left(P_{i},P_{T}\right)<1,\\ False,else, \end{array}\right. & if\;step<N_{Train}\text{,}\\ True\text{,} & else\text{,} \end{array}\right. \end{array}$$

where *N*_Train_ denotes the maximum iteration value to limit the number of times the agent implements the target point location operation in the environment during training stage, and its value is empirically set to 100. In the testing stage, because the ground truth of the target point cannot be provided, we design the following termination rule according to the experimental observation:

(3) $$\begin{array}{c} \delta_{Q}=abs\left(\frac{1}{n}\overset{i-8}{\underset{j=i-15}{\sum }}Q_{j}-\frac{1}{n}\overset{i}{\underset{k=i-7}{\sum }}Q_{k}\right)\text{,}\\ T_{test}=\left\{\begin{array}{cc} \left\{\begin{array}{c} True,if\;\delta_{Q}<Tr\;and\;q=T_{q,}\\ False,else, \end{array}\right. & if\;step<N_{Test}\text{,}\\ True\text{,} & else\text{,} \end{array}\right. \end{array}$$

where *δ*_*Q*_ represents the difference between the average value of the first eight elements in the last 16 *Q* values and the average value of the last eight elements in the last 16 *Q* values. *Tr* is the threshold which is set to 0.3. *q* is used to confirm whether the agent has converged to the target point, and its corresponding threshold *T*_*q*_ is set to 2 in this paper according to the time cost and experimental observation. *N*_Test_ denotes the maximum iteration value to limit the number of times the agent implements the target point location operation in the environment during testing stage, and its value is empirically set to 60.

After the previous brief introduction and analysis, it is followed by the typical reinforcement learning paradigm (i.e., Q-Learning) [42]. In various RL scenarios, appropriate actions are the core to achieve effective and continuous interaction between agent and environment, the optimization process of which can be completed based on the state-action value function *Q* (*s*, *a*) [43]. By solving the following formula, the corresponding *Q* value can be obtained after implementing the corresponding action in each state, and then the best action can be selected depending on the highest long-term *Q* value.

(4) $$\begin{array}{c} Q_{i+1}\left(s,a\right)=E\left[R+\gamma \max_{a^{\prime }}Q_{i}\left(s^{\prime },a^{\prime }\right)\right]\text{,} \end{array}$$

where *Q*_*i*+1_ and *Q*_*i*_ represent the *Q* values at step *I* and *i* + 1, respectively. *s* and *s*′ denote the current state and the next state, respectively. Correspondingly, *a* and *a*′ are the current action and the next action, respectively. *γ* is the discount factor and is set to 0.95 in this paper. However, when there are too many state-action pairs in Q-learning, the Q-Table will be too large and cause excessive memory consumption. At this time, the *Q*(*s*, *a*; *θ*) obtained by approximating *Q*(*s*, *a*) with the help of the deep neural network (i.e., Deep Q-Network, DQN) can avoid the previous issue. By periodically assigning the weight parameters of the current Q-Network to the target Q-Network, the parameters of the Q-Network can be continuously updated. Specifically, the parameters of DQN can be obtained by solving the following equation:

(5) $$\begin{array}{c} L_{DQN}\left(\theta \right)=E\left[{\left(R+\gamma \max_{a^{\prime }}Q\left(s^{\prime },a^{\prime };\theta^{\prime }\right)-Q\left(s,a;\theta \right)\right)}^{2}\right]\text{,} \end{array}$$

where *θ*′ and *θ* are the parameters of target Q-Network and the current Q-Network, respectively. In this paper, the DQN structure is designed as shown in Figure 6(b). The input size of the network is 32 × 32 × 4. During the training or testing stage, the input image is composed of four cropped single channel patches with the size of 32 × 32. After a series of convolutional layers, pooling layers, fully-connected layers, and various activation functions, a four-dimensional *Q*-value vector can be obtained, and then the corresponding action can be selected for the agent to interact with the environment.

#### 3.3.3. ILM-SARLM

**Figure 7:**
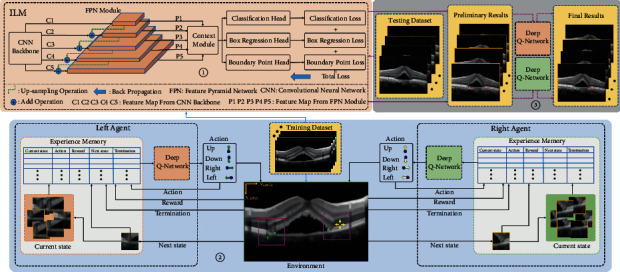
The overall framework of ILM-SARLM.

On the basis of the previous research, the joint framework shown in Figure 7 is finally established, mainly consisting of three parts, namely, ILM part, SARLM part, and joint location part. The execution contents of each part are as follows:

① ILM is firstly trained based on all the OCT B-scan images in the training dataset. Since five CNN backbone networks are applied in the ILM framework, the training process needs to be repeated five times. Then, offline testing models corresponding to various backbone networks can be acquired.
② It should be pointed out that the location model of key boundary points on both sides of the CSCR lesion area is trained independently. Although the network structure on both sides is the same, the weight parameters will not be shared between them. Under the previous premise, SARLMs are trained based on some OCT B-scan images in the training dataset to obtain independent DQN models for the action selection of left and right agents, respectively.
③ On the basis of ① and ②, the location task of key boundary points is achieved through a cascade operation. Specifically, the testing image is firstly sent to the offline testing model to obtain the initial coordinates of the key boundary points on both sides. Then, the testing image and its corresponding initial location results are sent to the DQN models, and the agents further optimize the initial points of ILM again in the purple square active region (as shown in Figure 7) which is delimited according to the initial points on both sides.

### 3.4. The Backbone Networks and Algorithm Step

This section shows the structures of five CNN backbone networks used in ILM, and the specific details are shown in Table 1. In addition, the algorithm steps of ILM are detailed in [18], and the algorithm steps of SARLM and joint location parts are shown in Table 2.

## 4. Experimental Settings

In order to verify the feasibility and effectiveness of the proposed scheme, this paper has conducted in-depth and extensive experiments. The training dataset, testing dataset, parameter settings and equipment conditions, and evaluation metric involved in the experiments will be briefly described.

### 4.1. Parameter Settings and Equipment Conditions

Both ILM and SARLM are based on the TensorFlow framework as the development platform, and the computing power involved in the training and testing process of these models is mainly supplied by NVIDIA-3080ti GPU. For ILM, the parameters of epoch, learning rate, batch size, and optimizer are set to 40, 0.001, 20, and Adam, respectively; [18]can be referred for other settings. For SARLM, the parameters of epoch, learning rate, batch size, max episode, update frequency, sample step, and optimizer are set to 80, 0.0001, 32, 25, 50, 5, and Adam, respectively.

### 4.2. Evaluation Metric

In order to quantify the location accuracy of the key boundary points in the CSCR lesion area so as to evaluate the performance of the proposed scheme, this paper adopts an average Euclidean distance (AED) evaluation metric, which is expressed as follows:

(6) $$\begin{array}{c} AED=\frac{1}{2N_{T}}\overset{N_{T}}{\underset{i=1}{\sum }}\left({\left\|\text{LG}\right\|}_{2}+{\left\|RG\right\|}_{2}\right)\text{.} \end{array}$$

For left key point,

(7) $$\begin{array}{c} \text{LG}=\left(x_{new\_L}-x_{truth\_L},y_{new\_L}-y_{truth\_L}\right)\text{.} \end{array}$$

For right key point,

(8) $$\begin{array}{c} RG=\left(x_{new\_R}-x_{truth\_R},y_{new\_R}-y_{truth\_R}\right), \end{array}$$

where *LG* and *RG* are vectors determined by the coordinate of the left key point (i.e., (*x*_new_*L*_, *y*_new_*L*_)), its corresponding ground truth value (i.e., (*x*_truth_*L*_, *y*_truth_*L*_)), the coordinate of the right key point (i.e., (*x*_new_*R*_, *y*_new_*R*_)), and its corresponding truth value (i.e., (*x*_truth_*R*_, *y*_truth_*R*_)), respectively. ‖*·*‖_2_ represents the 2-norm to perform the calculation of Euclidean distance, and *N*_*T*_ denotes the number of OCT B-scan images included in a single patient data. The diameter measurement of the CSCR lesion area is also based on the 2-norm, and the specific equation is as follows:

(9) $$\begin{array}{c} D={\left\|LR\right\|}_{2}\text{,} \end{array}$$

(10) $$\begin{array}{c} LR=\left(x_{new\_R}-x_{new\_L},y_{new\_R}-y_{new\_L}\right)\text{,} \end{array}$$

where *LR* is the vector determined by the left and right key boundary points.

## 5. Results and Discussions

This section analyzes and discusses the experiment. Before that, we will explain the terms involved in this process. It should be pointed out that in order to verify the effectiveness and feasibility of the joint framework proposed in this paper, not only the pure DQN based SARLM is combined with each kind of ILMs but also the DDQN-based [44, 45], Duel DQN (DuelDQN)-based [46], and Duel DDQN (DuelDDQN)-based SARLMs are introduced into the joint framework. As mentioned earlier, the SARLM model for locating the key boundary points on both sides in this paper is independent of each other, and the training process is carried out separately. In this way, the left key point location model based on DQN is named DQN_LP, and the corresponding location model for the right key point is named DQN_ RP. According to the same rule, DDQN_LP, DDQN_RP, DuelDQN_LP, DuelDQN_RP, DuelDDQN_LP, and DuelDDQN_RP can be obtained, respectively. Moreover, for different ILMs, we name it according to the name of the CNN backbone network in Section 3.3.1. We take the R18 backbone network and DQN for example, the pure ILM is named R18-Base, and the corresponding joint framework is called R18-DQN. The names of other joint frameworks also comply with this rule.

### 5.1. Convergence Observation of ILMs and SARLMs on the Training Dataset

**Figure 8:**
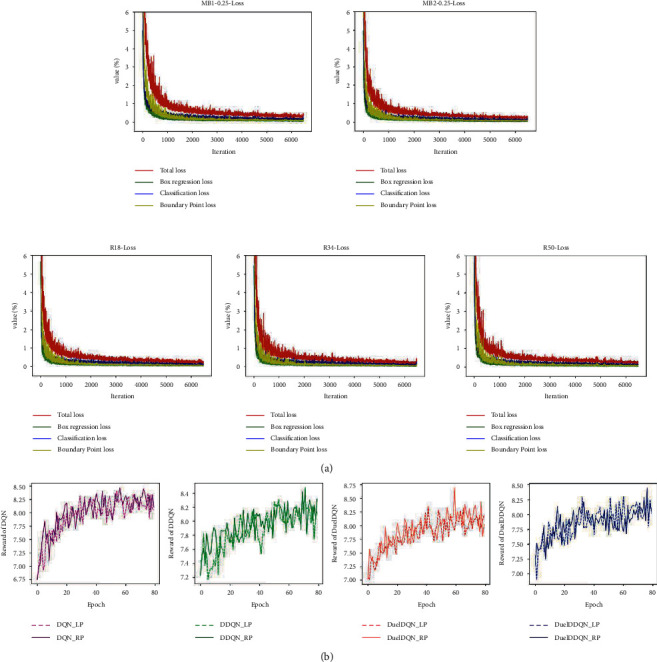
Convergence curves of ILM and SARLM in the training process: (a) the loss curves of different ILMs [16]; (b) the reward curves of different SARLMs.

Properly judging whether the model converges in the training process is an indispensable link related to the later model performance test and actual deployment. As shown in Figure 8(a), each ILM can converge from a large value to a small loss value after 6480 iterations, revealing that the weight parameters of the model can better fit a nonlinear function to deal with the task given in this paper. In addition, it can also be found that the progress trend of each task loss curve in ILM is almost the same, and the difference between losses is very small, which has important reference significance for setting the proportion parameter of each task item in the total loss. For SARLM, each model was trained with 80 epochs on the training dataset including 30 OCT B-scan images, and the total training time of eight models was about 240 hours, that is, 10 days. In order to boost the adaptability of SARLM to the initial position during the testing stage, the initial coordinate of the agent is initialized randomly in each episode of training, based on the coordinate ground truth of each key boundary point and the corresponding margin randomly selected from [−4, −6, −8, −10, −12, −14, 4, 6, 8, 10, 12, 14]. It can be clearly observed from Figure 8(b) that compared with the initial period, the reward value of each SARLM in the later training period finally stabilized within a certain range, which implies that through continuous exploration and exploitation, the agent gradually learned how to formulate appropriate behaviour strategies according to its environment to achieve the location task of the key boundary points in the CSCR lesion area. The previous observations show that the feasibility of ILMs and SARLMs in the training dataset has been preliminarily verified, laying a foundation for subsequent analysis.

### 5.2. Performance Analysis of SARLMs on the Validation Dataset

**Figure 9:**
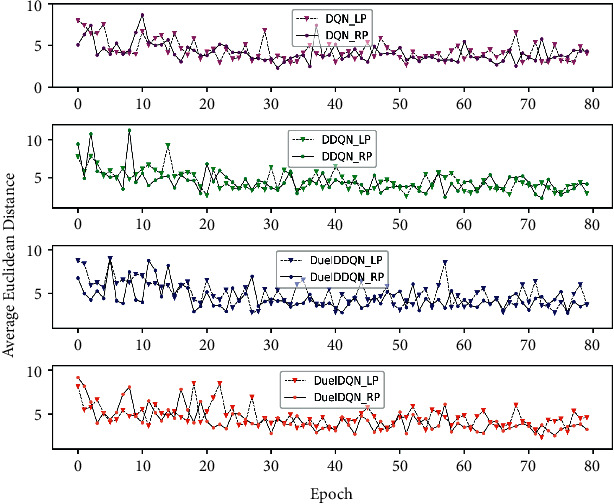
The AED of SARLMs under different epochs.

In order to select the appropriate SARLM for later application in the testing dataset, we conducted relevant experiments on the validation dataset consisting of 10 OCT B-scan images. Specifically, in order to check the location performance of SARLM under the random starting point in the local region of CSCR lesion, we designed 16 random initialization starting points for both the left and right agents based on the coordinate ground truth of the key boundary points and their corresponding margins in the *X*-axis direction and *Y*-axis direction (i.e., [−4, −14, 4, 14] for the *X*-axis direction and [−4, −14, 4, 14] for the *Y*-axis direction). Under the previous settings, the AED curves of various SARLMs based on 80 epochs are obtained as shown in Figure 9. On the whole, in the later stage of training, the AED value of each SARLM on the validation dataset decreases compared with the initial stage, which indicates that the ability of the agent to locate the key point under the condition of random initial position is appropriately improved with the increase of learning times, corresponding to the hint of the reward convergence curve of SARLM on the training dataset. Moreover, the overall trend of the AED curves of SARLMs for the left and right key points in the same environment is similar, indicating that even if the local regions of the key points on both sides are different, the strategy of training the SARLMs on both sides independently in this paper can make the agents properly adapt to this change.

**Figure 10:**
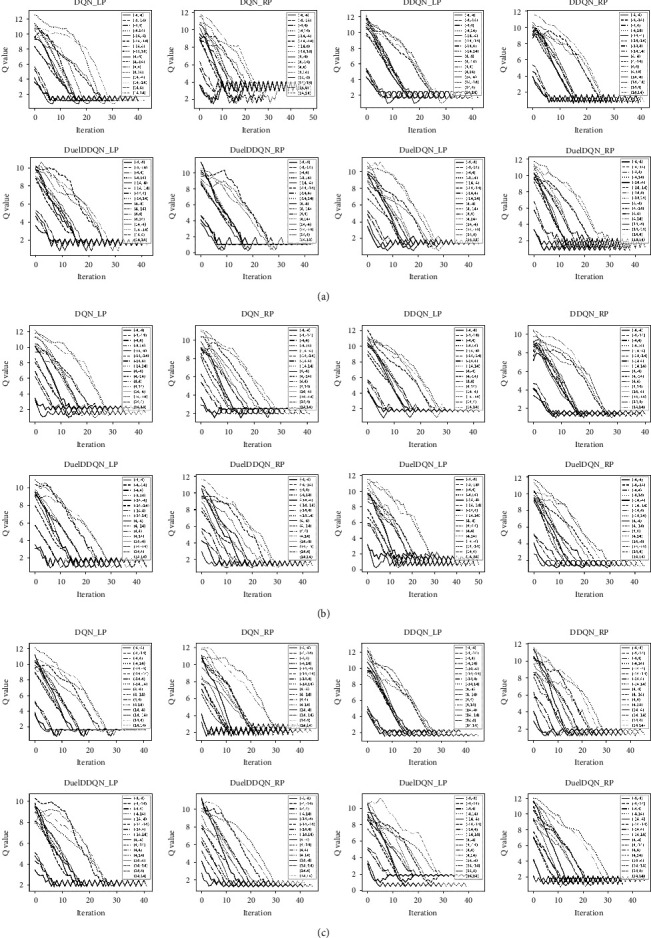
*Q* value convergence curve of SARLMs under different initial starting points: (a) example-1; (b) example-2; (c) example-3.

*Q* value is an important indicator to measure the closeness of agents to target points and is the key basis to terminate the interaction between agents and the environment. As pointed out in [29], when the agent approaches the target point, *Q* value is relatively small; otherwise, it is relatively large. This view is further confirmed by the experimental results involved in the task of this paper. As shown in Figure 10, each best SARLM-selected based on the minimum AED can almost converge from the larger *Q* value at the initial time to the smaller *Q* value at the final stage, which means that the agent can perform well in the validation dataset after continuous learning. In some cases, when the initial position of the agent is set to approach the target point, the *Q* value does not change much before and after convergence, suggesting that the agent judges that it is close to the target point fall into the local optimized point in advance. This situation is also where we strive to break in the future.

**Figure 11:**
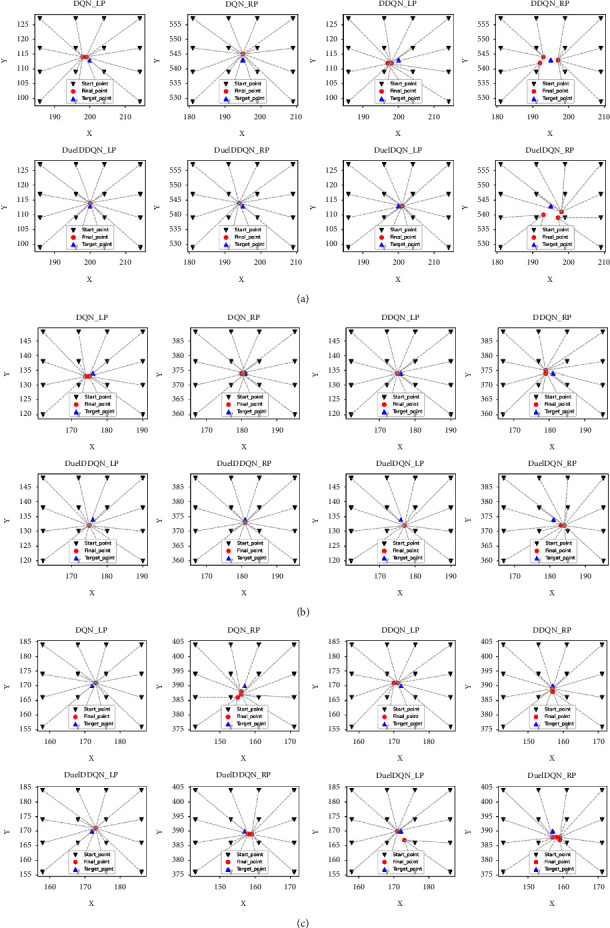
The location of key boundary points of SARLMs under different initial starting points: (a) example-1; (b) example-2; (c) example-3.

However, we can be gratified that when the initial position of the agent is far away from the target point, each kind of SARLM can stabilize at a small *Q* value after the iteration stops, which shows that even if the maximum distance between the agent and the target point is about 19 pixels in the local region, it can finally converge near the target point. In fact, the initial location accuracy of ILM is usually less than this value (as shown in Table 3), which proves that it is feasible to use ILM to narrow the active region of the agent for SARLM. Corresponding to Figures 10 and 11, it shows the visual performance of each SARLM in the key boundary point location task under 16 initial positions, in which the silver dotted line represents the final position of the agent corresponding to the initial position. What can be clearly captured is that the agent can finally converge near the target point at different initial positions. Although there are great differences in each initial position, each final position of the agent is very close, and in some cases, the agent even locks the same final point (such as Figure 11(a) DQN_RP, DuelDDQN_LP, DuleDDQN_RP and so on). The previous analysis not only shows the good location ability of each SARLM on the validation dataset but also implies its low sensitivity to the initial position in the local region, which plays an important role in promoting the proposal of the joint framework.

### 5.3. Performance of ILMs and ILM-SARLMs on the Testing Dataset

Based on the previous results and analysis, this section formally investigates the performance of the joint framework constructed based on the multitask learning and the single-agent reinforcement learning on the testing dataset, including qualitative analysis, quantitative analysis, and efficiency analysis, so as to explore and discover the value and potential of the framework in practical application scenarios and the corresponding details to be further improved.

#### 5.3.1. Qualitative Analysis

**Figure 12:**
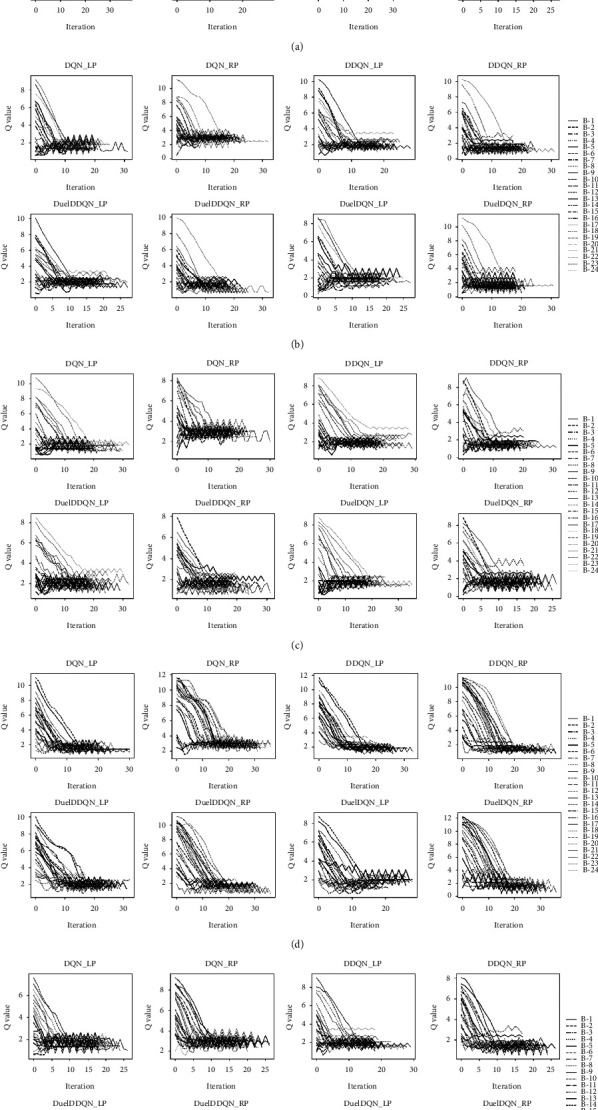
*Q* value convergence curve of SARLMs under different ILMs: (a) MB1-0.25 (b) MB2-0.25 (c) R18 (d) R34 (e) R50.

**Figure 13:**
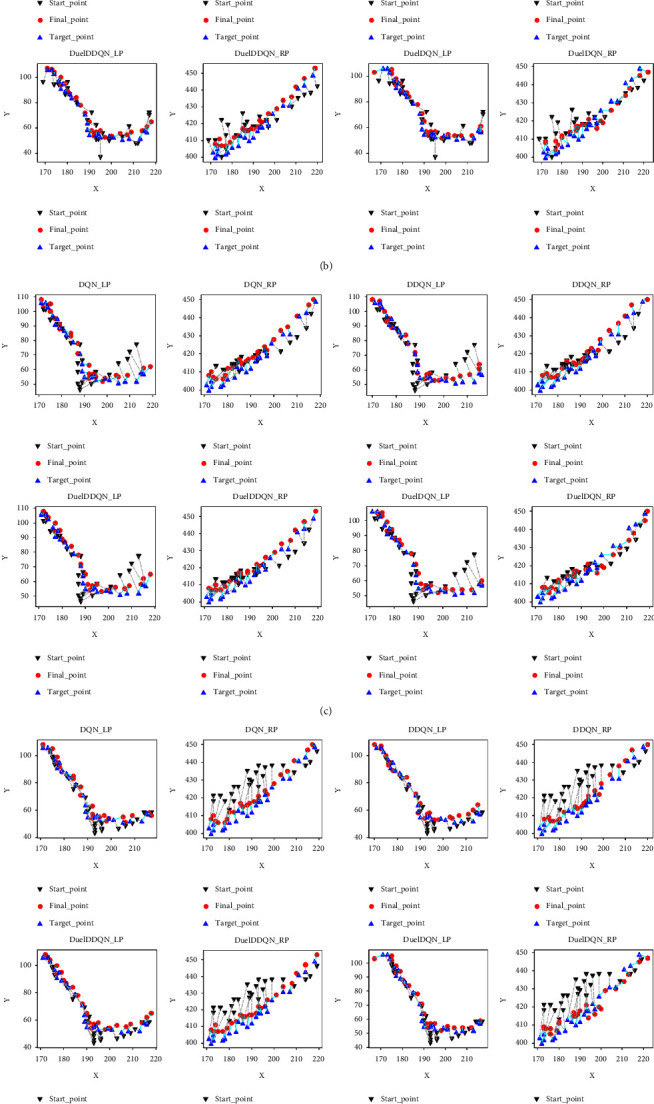
The location of key boundary points of SARLMs under different ILMs: (a) MB1-0.25; (b) MB2-0.25; (c) R18; (d) R34; (e) R50.

As shown in Figures 12 and 13, the *Q* value convergence and practical application effect of the joint framework composed of different ILMs and SARLMs under the key boundary point location task at the image level are shown, respectively. The icon on the right in Figure 12 represents the scanning number of all B-scan images in the patient-level data. In general, each joint framework can achieve convergence under a certain number of iterations, that is, obtain a smaller *Q* value in the later stage. However, similar to the observations in the validation dataset, the difference between the *Q* value of the agent before and after the key point location task in some B-scan images of the testing dataset is small. This reveals that the initial starting point provided by ILM for SARLM is relatively close to the target point, thus making the agent wander around the target point all the time, and the external representation is the periodic small amplitude fluctuation of *Q* value. As shown in Figure 13, the reason behind the previous phenomenon is further demonstrated by the actual location effect. It can be clearly captured that sometimes the initial starting point is indeed in the region near the target point, resulting in the agent naturally interacting with the environment in a small range and gaining a small *Q* value, which echoes the observation in Figure 12. In addition, the cyan line in the figure represents the distance between the final location point of the joint framework and the target point, while the silver dotted line here denotes the distance between the starting point provided by ILM and the target point. It can be seen that in most cases, the length of the cyan line is smaller than that of the silver dotted line, which indicates that the joint framework has the ability to further optimize the position of the initial key boundary points. Meanwhile, Figure 13 also visually shows the performance difference between joint frameworks and conveys the level of various ILMs in the initial location task, which is conducive to the selection and deployment of the joint framework in practical application.

**Figure 14:**
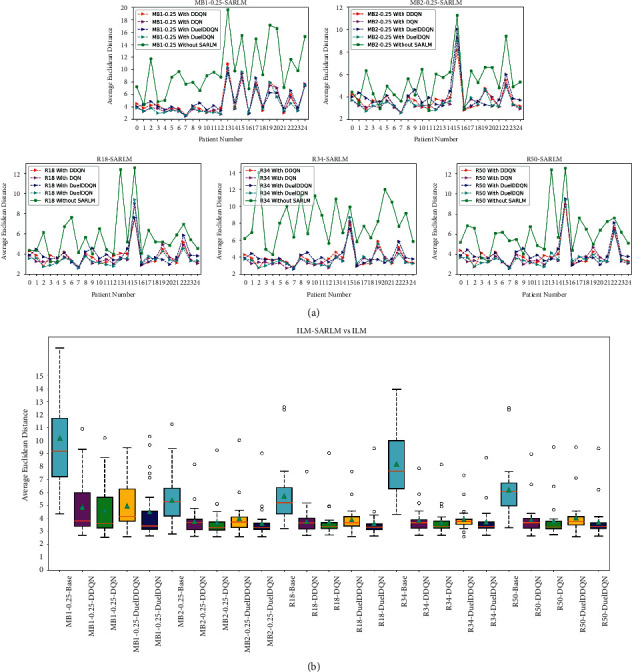
The performance of ILM-SARLMs under patient level: (a) the AED difference between models; (b) the robustness difference between models.

In addition to the previous qualitative analysis at the picture level, we also verify the ability of the different joint framework and the corresponding ILMs on patient-level data, so the discussion based on patient level is also introduced here.  Figure 14(a) clearly reveals the AED of each ILM and its corresponding joint framework on each patient-level data. It is obvious that compared with the pure ILMs, all kinds of joint frameworks can basically obtain lower AED values, further enriching the evidence of the effectiveness of the proposed model. Furthermore, the robustness of various joint frameworks and their ILMs at the patient level has also been carefully considered. As shown in Figure 14(b), compared with ILM, the box height of its corresponding joint framework is relatively small, showing that the performance of the joint framework on patient-level data fluctuates little, implying that the joint framework has good robustness. The cyan triangle in the figure denotes the average AED value of ILM or joint framework. The lower value also proves that the joint framework possesses a more prominent key point location ability as a whole. What can also be seen is that there is a significant difference in robustness between joint frameworks, which, together with the information provided in Figure 13, sets a reference for the selection of models.

**Figure 15:**
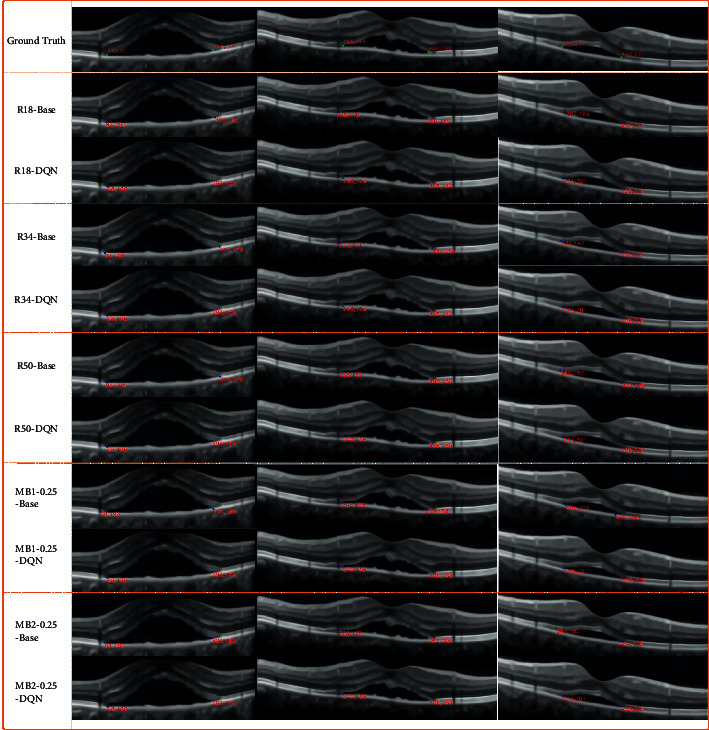
The display of location results of SARLMs under different ILMs.

At the end of this section, the DQN-based SARLM is taken as an example to show the actual results of the joint frameworks under different ILMs in locating the key boundary points of CSCR lesion. As shown in Figure 15, with the ground truth as a reference, it is easily judged that various joint frameworks can achieve the effective reoptimization of the position of key boundary points on the basis of the initial results of ILM, which is consistent with the previous qualitative analysis based on the AED metric. Through the actual location results, the effectiveness and feasibility of the joint framework are demonstrated again.

#### 5.3.2. Qualitative Analysis

**Figure 16:**
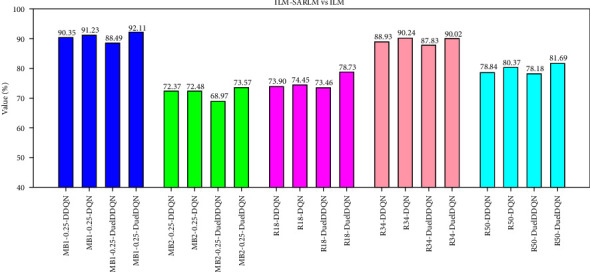
The successful correction ratio of key boundary points of ILM-SARLMs under image level.

This section further analyzes the performance between the joint framework and the corresponding ILM from the quantitative perspective. When the AED between the key boundary points on both sides provided by the joint framework and the corresponding target points is less than that between the key boundary points on both sides provided by the ILM and the corresponding target points, the joint framework successfully corrects the key boundary points on the B-scan image. As shown in Figure 16, from the image level, the successful correction rate of the key boundary point of the joint framework exceeds half of the total number of testing images, and the maximum value reaches 92.11% (i.e., a total of 840 B-scan images). Besides, there are some differences in the successful correction rate between joint frameworks based on five kinds of ILMs. However, in view of the defects of the weak generalization ability of ILM itself and the performance differences between them, it cannot be directly said that a smaller value corresponds to a joint framework with weak correction ability, and a larger value represents a strong ability. The successful correction rate here mainly reflects that the joint framework can further optimize the location of key boundary points based on the corresponding ILM. As for the comparison of performance differences between models, it can be based on the qualitative analysis in the previous section and the AED metric at the patient level shown in Table 3.

As shown in Table 3, the patient-level-based AEDs are counted. It can be clearly observed that each joint framework is significantly superior to the corresponding ILM in terms of this metric, obtaining an AED value with a minimum of 3.61 pixels, and the maximum difference between the two types of models is 5.68 pixels, which quantitatively shows the advantages of the joint framework. Additionally, due to the introduction of SARLM, the size of the joint framework is larger than that of the corresponding ILM, but this margin has little impact on the actual deployment. In general, the previous quantitative analysis also confirms the positive role of the joint framework in the given task.

#### 5.3.3. Efficiency Analysis

**Figure 17:**
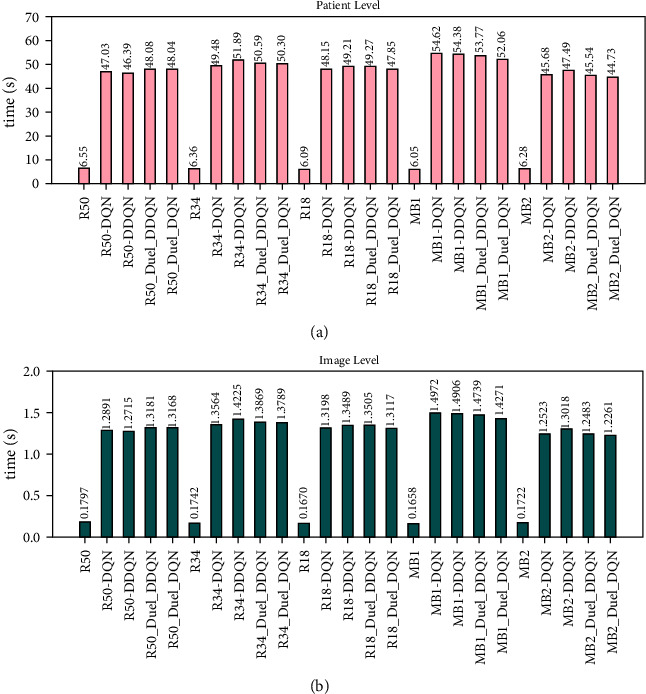
Comparison of time consuming between ILMs and ILM-SARLMs: (a) average time consuming at patient level; (b) average time consuming at image level.

This section focuses on analyzing the efficiency of the proposed joint framework and ILM. The time cost is the key reference to judge the efficiency of the model in completing the given task. In view of this, we recorded the time cost of ILMs and the corresponding joint frameworks in the key boundary point location task in the CSCR lesion.  Figure 17(a) shows the time consumption based on the patient level, in which the time of ILM is significantly lower than that of the joint framework, and its minimum time consumption is only about 6.05 seconds. Furthermore, the time consumption based on the image level reflected in Figure 17(b) also displays the efficiency advantage of various ILMs. This situation is mainly due to the fact that SARLM in the joint framework needs to rely on the continuous interaction between the agent and the environment. Such an iterative process will naturally lead to an increase in time cost. To alleviate the previous issue, this paper aims to compress the time expenditure for the joint framework from two perspectives. On the one hand, the active region of the agent is limited to a local scope through the initial key boundary point location function of ILM, so as to avoid the transition time consumption caused by the global activities of the agent. On the other hand, the termination rule is properly developed to stop the repeated interaction behaviour of the agent in the later stage as soon as possible when it has approached the target point. The former can undoubtedly improve the efficiency of the agent. As for the latter, although the termination rule based on the *Q* value and the maximum iteration designed in this paper can appropriately stop the repeated wandering behaviour of the agent on time, better termination hints still need to be explored, which is also the perspective we need to further improve in the future.

### 5.4. Discussion on the Effectiveness of SARLMs for ILM-GBCMs on the Testing Dataset

**Figure 18:**
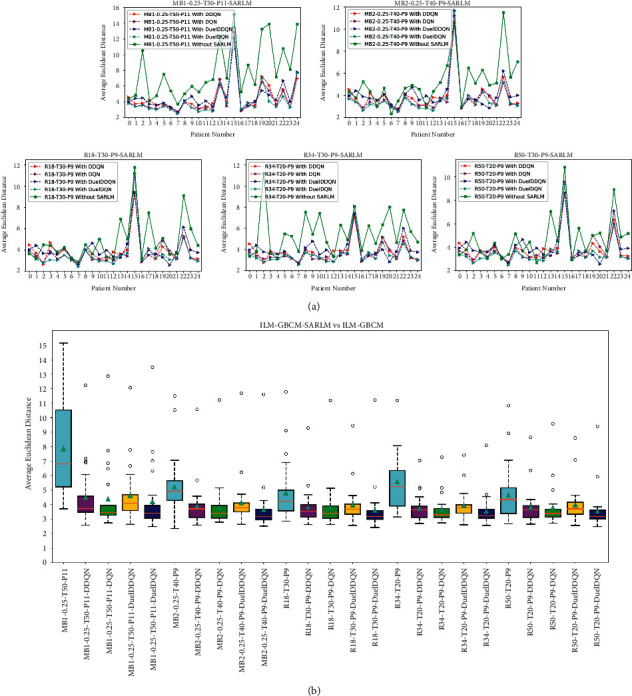
The performance of ILM-GBCM-SARLMs under patient level: (a) the AED difference between models; (b) the robustness difference between models.

The analysis and discussion in the previous sections show the superiority of the joint framework proposed in this paper over ILM. This section intends to explore whether SARLM can be equally effective in further improving the performance of ILM-GBCM [18] through qualitative and quantitative experiments. As shown in Figure 18, MB1-0.25-T50-P11, MB2-0.25-T40-P9, R18-T30-P9, R34-T20-P9, and R50-T20-P9 represent different ILM-GBCMs, respectively. Similarly, taking the DQN-based SARLM as an example, the joint frameworks based on the previous GBCMs are MB1-0.25-T50-P11-DQN, MB2-0.25-T40-P9-DQN, R18-T30-P9-DQN, R34-T20-P9-DQN, and R50-T20-P9-DQN, respectively.  Figure 18(a) shows that the AED values of the joint frameworks at the patient level are higher than those of the corresponding ILM-GBCMs most of the time, indicating that the joint framework can further optimize the position of the key boundary points after the GBCM correction. It can also be found from Figure 18(b) that whether considering the average value of AED (the cyan triangle in the figure) or based on the box height, all kinds of joint frameworks are significantly better than their corresponding ILM-GBCMs, not only confirming the role of the joint framework in improving the location accuracy of key boundary points of ILM-GBCM but also revealing its good robustness in this task.

**Figure 19:**
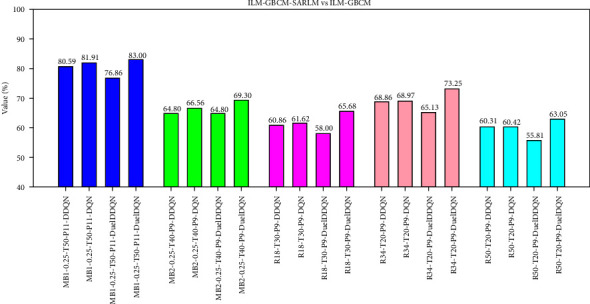
The successful correction ratio of key boundary points of ILM-GBCM-SARLMs under image level.

Furthermore, quantitative analysis experiments were carried out accordingly. As shown in Figure 19, the maximum successful correction rate of the joint framework is 83.00%, which shows that it has the ability to further improve the location accuracy on the basis of the correction results. However, by observing Figures 16 and 19, it can be found that compared with the successful correction rate of the location results of pure ILM, the improvement degree of SARLM on this metric of ILM-GBCM is relatively low, which stems from the fact that GBCM has played a corrective role in the initial location results of ILM to a certain extent, thus making the space for SARLM to further optimize the location accuracy of key boundary points smaller. Concurrently, AEDs of the two models based on the patient level are also emphatically recorded, as shown in Table 4. Compared with ILM-GBCM, the AED values of the corresponding ILM-GBCM-SARLM are reduced, and the maximum difference between them is 3.64 pixels, which together with the image-level-based successful correction rate shows that SARLM also has a certain effect in boosting the ability of ILM-GBCM to locate the key boundary points in the CSCR lesion area.

### 5.5. Preliminary Application

**Figure 20:**
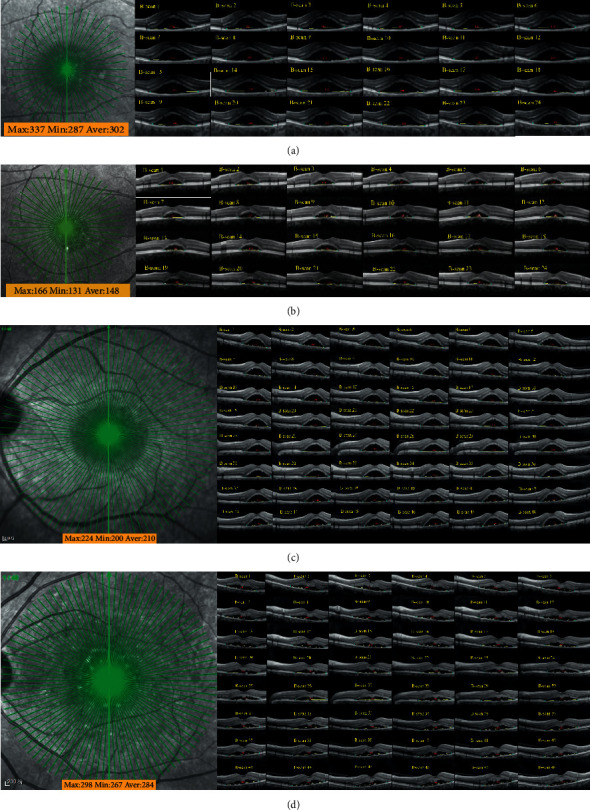
The diameter measurement of CSCR lesion area based on ILM-SARLM: (a) example-1; (b) example-2; (c) example-3; (d) example-4.

Extensive experiments and analysis have proved the superiority of the proposed joint framework compared with the corresponding ILM in the key boundary point location task in the CSCR lesion area, which is embodied in the fact that it can make up for the weak generalization of ILM in this scene through the unique autonomous learning ability of agent in the lesion environment, which paves the way for its preliminary application in the actual measurement of the diameter of CSCR lesions in this section. Based on formulas (9) and (10) and the coordinates of the key boundary points output by the joint framework, the diameters of the CSCR lesions at all scanning angles can be measured quickly. As shown in Figure 20, the diameter measurement results of four patient-level samples, in which Figures 20(a) and 20(b) correspond to samples containing 24 OCT B-scan images, and Figures 20(c) and 20(d) correspond to samples containing 48 OCT B-scan images. On this basis, it is not only convenient for ophthalmologists to review the diameter size of the lesion at all scanning angles but also convenient for them to check the diameter size of the lesion at a certain scanning angle. Moreover, based on the diameter measurement results of lesions in all B-scan images, the maximum, minimum, and average values can also be obtained, providing a quantitative reference for ophthalmologists to judge the severity of the CSCR and evaluate the efficacy of the corresponding treatment scheme.

### 5.6. The Clinical Advantages of This Study in Ophthalmology

By and large, in the previous sections, both the qualitative-quantitative analysis oriented to the evaluation of location effect and the time-cost consideration oriented to the evaluation of efficiency provide strong support for demonstrating the feasibility, effectiveness, and potential clinical application value of the proposed joint framework based on ILM and SARLM in the task of locating key boundary points in the CSCR lesion area. In addition, the qualitative-quantitative experiments of the joint framework composed of SARLM and ILM-GBCM reveal that the introduction of SARLM can also add luster to the further optimization of the initial key boundary points provided by ILM-GBCM. On the basis of the previous part, based on the preliminary application of the diameter measurement of the CSCR lesion area, the potential deployment value of the proposed joint framework is affirmed from a practical point of view, and the significance of this study is further consolidated. It should be noted that although the scheme proposed in this paper is aimed at the diameter measurement of the CSCR lesion area, this method is also instructive for the design of the automatic measurement scheme of the diameter of the focus area in OCT B-scan images of other fundus diseases (such as diabetic macular edema, macular hole, and retinal angiomatous proliferation), which has potential guiding significance for more comprehensive monitoring of the lesion morphology and assisting ophthalmologists in the more objective assessment of patients' eye conditions.

### 5.7. Limitation Analysis and Future Work

Although this study reveals good clinical application value, the inadequacy of the proposed scheme still needs to be properly faced. Specifically, the joint framework may encounter the phenomenon of an agent wandering around the target point repeatedly in the procedure of processing a given task, which is also the factor leading to its slightly lower efficiency. We hold that this phenomenon may be due to the fact that the surrounding environment is too similar when the agent is approaching the target point in the process of interaction with the environment, resulting in the agent being unable to learn more effective behavior strategies and then falling into a local area and unable to extricate itself. For this problem, in the future, we plan to continue to mine solutions from the design of the termination rule and the adjustment of the DQN framework structure, so as to further improve the performance of the joint framework.

## 6. Conclusions

An automatic image processing method (i.e., the joint framework) based on the multitask learning and single-agent reinforcement learning paradigms is constructed to achieve the goal of rapid and accurate location of key boundary points in CSCR lesion area, so as to facilitate the automatic diameter measurement of CSCR lesion. On the one hand, the adjustment and introduction of ILM initially realize the rapid locking of key boundary points and effectively narrow the activity range of agents, which helps to improve the location efficiency of SARLM. On the other hand, the unique exploration ability of the agent enables it to independently learn task-oriented behavior strategies, so that SARLM can better adapt to the differences of CSCR lesion areas in different scanning frames and appropriately make up for the defect of low generalization of ILM. Extensive experiments have been carried out carefully, demonstrating the effectiveness and feasibility of the joint framework in improving the location performance of ILM. The preliminary test on the diameter measurement of the CSCR lesion further reveals the potential clinical application value of the proposed joint framework, which also has a certain reference significance for the designation of the diameter measurement scheme of lesions in other fundus diseases (diabetic macular edema, macular hole, and retinal angiomatous proliferation). Generally speaking, the method proposed in this paper is a further innovation based on our previous work from the perspective of the algorithm, and in the future, we will pay attention to the inadequacy of this scheme and improve it.

## Figures and Tables

**Table 1:** The five backbone networks used in ILM.

Layer name	Output size	R18	R34	R50	MB1-0.25	MB2-0.25
Conv1	C5: 160 × 160	7 × 7, 64, stride 2			3 × 3, 8, stride 2	3 × 3, 8, stride 2
					3 × 3, 8	1 × 1, 48
					1 × 1, 16	3 × 3, 48
						1 × 1, 16

Conv2	C4: 80 × 80	3 × 3 max pool, stride 2			3 × 3, 16, stride 2	1 × 1, 96
		$\left[\begin{array}{cc} 3\times 3\text{,} & 64\\ 3\times 3\text{,} & 64 \end{array}\right]\times 2$	$\left[\begin{array}{cc} 3\times 3\text{,} & 64\\ 3\times 3\text{,} & 64 \end{array}\right]\times 3$	$\left[\begin{array}{cc} 1\times 1\text{,} & 64\\ 3\times 3\text{,} & 64\\ 1\times 1\text{,} & 256 \end{array}\right]\times 3$	1 × 1, 32	3 × 3, 96, stride 2
					3 × 3, 32	1 × 1, 32
					1 × 1, 32	1 × 1, 192; 3 × 3, 192
						1 × 1, 32

Conv3	C3: 40 × 40	$\left[\begin{array}{cc} 3\times 3\text{,} & 128\\ 3\times 3\text{,} & 128 \end{array}\right]\times 2$	$\left[\begin{array}{cc} 3\times 3\text{,} & 128\\ 3\times 3\text{,} & 128 \end{array}\right]\times 4$	$\left[\begin{array}{cc} 1\times 1\text{,} & 128\\ 3\times 3\text{,} & 128\\ 1\times 1\text{,} & 512 \end{array}\right]\times 4$	3 × 3, 32, stride 2	1 × 1, 192
					1 × 1, 64	3 × 3, 192, stride 2
					3 × 3, 64	1 × 1, 64
					1 × 1, 64	1 × 1, 384; 3 × 3, 384
						1 × 1, 64

Conv4	C2: 20 × 20	$\left[\begin{array}{cc} 3\times 3\text{,} & 256\\ 3\times 3\text{,} & 256 \end{array}\right]\times 2$	$\left[\begin{array}{cc} 3\times 3\text{,} & 256\\ 3\times 3\text{,} & 256 \end{array}\right]\times 6$	$\left[\begin{array}{cc} 1\times 1\text{,} & 256\\ 3\times 3\text{,} & 256\\ 1\times 1\text{,} & 1024 \end{array}\right]\times 6$	3 × 3, 64, stride 2	1 × 1, 384
					1 × 1, 128	3 × 3, 384, stride 2
					$\left[\begin{array}{cc} 3\times 3\text{,} & 128\\ 3\times 3\text{,} & 128 \end{array}\right]\times 5$	1 × 1, 128
						$\left[\begin{array}{cc} 1\times 1\text{,} & 768\\ 3\times 3\text{,} & 768\\ 1\times 1\text{,} & 128 \end{array}\right]\times 5$

Conv5	C1: 10 × 10	$\left[\begin{array}{cc} 3\times 3\text{,} & 512\\ 3\times 3\text{,} & 512 \end{array}\right]\times 2$	$\left[\begin{array}{cc} 3\times 3\text{,} & 512\\ 3\times 3\text{,} & 512 \end{array}\right]\times 3$	$\left[\begin{array}{cc} 1\times 1\text{,} & 512\\ 3\times 3\text{,} & 512\\ 1\times 1\text{,} & 2048 \end{array}\right]\times 3$	3 × 3, 128, stride 2	1 × 1, 768
					1 × 1, 256	3 × 3, 768, stride 2
					3 × 3, 256	1 × 1, 256
					1 × 1, 256	1 × 1, 1536; 3 × 3, 1536
						1 × 1, 256

**Table 2:** The algorithm of ILM-SARLM.

**Input ** *D*_*Train*_, *N*_*Train*_, *γ*, learning rate, batch size *B*, epoch, max episode *P*, update frequency *L*, sample step *S*;
^*∗*^ **Training step for SARLM** ^*∗∗*^
**//**Initialize *θ*, *θ*′, and Experience memory *M*;
**for ** *each K in* epoch **do**
**//**Shuffle *D*_*Train*_;
**//While** (*j* <= *D*_*Train*_); select the *j*^*th*^ B-scan image *B*^*j*^;
**//**Initialize Experience memory *M*;
**for ***each k inP ***do**
Initialize *T*_*train*_=*False* and *step*;
**While not***T*_*train*_**and ***step* *<* *N*_*Train*_;
**//**Agent interact with environment *E* using *a* selected randomly
or calculated by argmax_*a*_*Q*(*s*, *a*; *θ*), and receive next state *s*′;
**//**Get reward *R* using (1) and termination flag *T*_*train*_ using (2);
**//**Store (*s*, *a*, *R*, *s*′, *T*_*train*_) in *M*;
**if ***M* ≥ batch size and *M* % *S* ⩵ 0;
//Randomly select samples at size of *B* from *M*;
**if***T*_*train*_;
//Calculate *L*_DQN_(*θ*)=*E*[(*R* − *Q*(*s*, *a*, *θ*))^2^];
//Update *θ* using Adm optimizer;
//Update *θ*′ = *θ* every *L* steps;
**else**
//Calculate (5);
//Update *θ* using Adm optimizer;
//Update *θ*′ = *θ* every *L* steps;
**end**
**end**
**// ***step*++;
**end**
**// ***j ***++**;
**end**

**Input ** *D*_*Test*_, Tr, *N*_*Test*_, *T*_*q*_;

^*∗∗*^ **Testing step for ILM-SARLM** ^*∗∗*^
**//**Initialize *q* and *step*;
**//**Get the initial coordinates (*x*_*L*_, *y*_*L*_) and (*x*_*R*_, *y*_*R*_) using the offline testing ILM;
**//**Send the initial results and testing images into the DQN models;
**While** True;
//Agents interact with environment *E* using actions calculated by argmax_*a*_*Q*(*s*, *a*; *θ*);
//Calculate *δ*_*Q*_ using (3);
//*step*++;
**if ***step* **<** *N*_*Test*_ and *δ*_*Q*_ < *Tr*;
//*q*+ = 1;
**end**
//Calculate *T*_*test*_ using (3) and (4);
**if***T*_*test*_**or ***step* = **=** *N*_*Test*_;
//Agents stop interaction behaviour and output the final location coordinates
(*x*_new_*L*_, *y*_new_*L*_) and (*x*_new_*R*_, *y*_new_*R*_);
**Break**

**Table 3:** Comparison of ILMs and ILM-SARLMs based on the AED.

	Model	AED (pixels)	Size (M)
ILM-SARLMs	R18-DQN	3.71	166.2
	R18-DDQN	3.78	
	R18-DuelDQN	3.61	
	R18-DuelDDQN	3.91	
	R34-DQN	3.71	283.2
	R34-DDQN	3.88	
	R34-DuelDQN	3.72	
	R34-DuelDDQN	3.95	
	R50-DQN	3.79	313.2
	R50-DDQN	3.91	
	R50-DuelDQN	3.72	
	R50-DuelDDQN	4.06	
	MB1-0.25-DQN	4.61	41
	MB1-0.25-DDQN	4.87	
	MB1-0.25-DuelDQN	4.51	
	MB1-0.25-DuelDDQN	4.97	
	MB2-0.25-DQN	3.68	66.3
	MB2-0.25-DDQN	3.78	
	MB2-0.25-DuelDQN	3.62	
	MB2-0.25-DuelDDQN	4.01	

ILMs	R18	5.73	138
	R34	8.21	255
	R50	6.21	285
	MB1-0.25	10.19	12.8
	MB2-0.25	5.40	38.1

**Table 4:** Comparison of ILM-GBCMs and ILM-GBCM-SARLMs based on the AED.

	Model	AED (pixels)	Size (M)
ILM-GBCM-SARLMs	R18-T30-P9-DQN	3.79	166.2
	R18-T30-P9-DDQN	3.82	
	R18-T30-P9-DuelDQN	3.61	
	R18-T30-P9-DuelDDQN	3.98	
	R34-T20-P9-DQN	3.63	283.2
	R34-T20-P9-DDQN	3.74	
	R34-T20-P9-DuelDQN	3.54	
	R34-T20-P9-DuelDDQN	3.93	
	R50-T20-P9-DQN	3.78	313.2
	R50-T20-P9-DDQN	3.81	
	R50-T20-P9-DuelDQN	3.56	
	R50-T20-P9-DuelDDQN	3.99	
	MB1-0.25-T50-P11-DQN	4.38	41
	MB1-0.25-T50-P11-DDQN	4.50	
	MB1-0.25-T50-P11-DuelDQN	4.21	
	MB1-0.25-T50-P11-DuelDDQN	4.64	
	MB2-0.25-T40-P9-DQN	3.79	66.3
	MB2-0.25-T40-P9-DDQN	3.91	
	MB2-0.25-T40-P9-DuelDQN	3.66	
	MB2-0.25-T40-P9-DuelDDQN	4.13	

ILM-GBCMs	R18-T30-P9	4.79	138
	R34-T20-P9	5.57	255
	R50-T20-P9	4.65	285
	MB1-0.25-T50-P11	7.85	12.8
	MB2-0.25-T40-P9	5.21	38.1

## Data Availability

The CSCR dataset used and analyzed in our research is available from the corresponding authors upon reasonable request.
